# 

**Published:** 1911-07

**Authors:** 


					﻿Vol. XXVIL
JUI/fciail.
• X‘o. ” T
I TIE X fXSi
MEDICAL
JOURNAL
“Red Back”
PUBLISHED AT AUSTIN, TEXAS, BY
F. E. DANIEL, M. D., - - - Editor
PRINTED BY THE VON BOECKXVNjQ|Kh%
Subscription, SI .00 a Year jin Advance.	-	-	-	- Single Copies, 10 Cents
Entered at tbe Postoffice at Austin. Tèsala* second class mail matter.
				

